# Prevalence of *Rickettsia* species phylotype G022 and *Rickettsia tillamookensis* in *Ixodes pacificus* nymphs and adults from Northern California^☆^

**DOI:** 10.1016/j.ttbdis.2025.102463

**Published:** 2025-03-19

**Authors:** Erin Trent, Andrea Swei, Tina Feiszli, Megan E.M. Saunders, Jianmin Zhong

**Affiliations:** a Department of Biological Sciences, Cal Poly Humboldt, Arcata, CA, USA; b Department of Biology, San Francisco State University, San Francisco, CA, USA; c Vector-Borne Disease Section, Infectious Diseases Branch, California Department of Public Health, Richmond, CA, USA

**Keywords:** *Ixodes pacificus*, Nymphal ticks, *Rickettsia* species phylotype G022, *Rickettsia tillamookensis*

## Abstract

Ticks are known vectors of various pathogenic bacteria, including species of *Rickettsia*. Two novel *Rickettsia* species have been identified in adult *Ixodes pacificus: Rickettsia* species phylotype G022 in 2011 and *R. tillamookensis* in 2021. Currently, the pathogenic potential of these species found in *I. pacificus* remains unknown. This study aimed to determine the prevalence of phylotype G022 and *R. tillamookensis* in *I. pacificus* nymphs across different mean annual temperature and relative humidity zones in California. Adult ticks were also tested for phylotype G022. Ticks were collected from multiple locations in seven northern California counties and tested by real-time PCR. The overall prevalence of phylotype G022 and *R. tillamookensis* in nymphs (*n* = 550) was 5.3 % (95 % CI = 3.7 %-7.5 %) and 1.6 % (95 % CI=0.8 %–3.3 %), respectively. The overall prevalence of phylotype G022 in adult *I. pacificus* (*n* = 720) was 9.0 % (95 % CI = 7.2 %-11.3 %). Phylotype G022 infects nymphal *I. pacificus* across a broad geographic range. The prevalence of phylotype G022 was higher in the 11.7–13.3 °C (53–56°F) temperature zone, at 6.4 % (95 % CI = 4.5 %-9.2 %), compared to the 13.9–15 °C (57–59°F) zone, where the prevalence was 0.8 % (95 % CI = 0.2 %-4.6 %). In contrast, the prevalence of *R. tillamookensis* did not show a statistically significant difference between the two temperature zones, with 1.9 % (95 % CI = 0.9 %-4.1 % in the 11.7–13.3 °C (53–56°F) zone and 0.9 % (95 % CI = 0.2 %-4.9 %) in the 13.9–15 °C (57–59°F) zone. The detection of phylotype G022 in both questing nymphs and adults of *I. pacificus* suggests that it is transmitted transstadially. qPCR testing revealed no coinfections of G022 and *R. tillamookensis* in any of the nymphs. Although *R. tillamookensis* exhibited a lower overall prevalence in nymphs compared to phylotype G022, both bacteria exhibited a similar geographic distribution.

## 1. Introduction

Ticks transmit the greatest variety of arthropod-borne pathogens that cause the most cases of vector-borne disease in the United States and Northern Hemisphere, second only to mosquitoes worldwide (Socolovschi et al., 2009; Eisen et al., 2017; Sonenshine and Macaluso, 2017; MacDonald, 2018; Swei et al., 2020). California is home to several medically important ticks including the western black-legged tick, *Ixodes pacificus* (Eisen et al., 2017; Furman and Loomis, 1984), the primary vector of pathogens causing Lyme borreliosis, human anaplasmosis, and *Borrelia miyamotoi* disease in the western United States. While nymphal and adult *I. pacificus* ticks transmit pathogens, the nymphs are thought to account for more disease transmission to humans and other vertebrates due to their smaller size and their peak questing activities coinciding with increased outdoor activity by people (Clover and Lane, 1995; Padgett and Bonilla 2011). The nymphal stage of *I. pacificus* has also been shown to have a higher burden of certain pathogenic bacteria, such as *Borrelia burgdorferi*, than the adult stage (Clover and Lane, 1995; Lane and Quistad, 1998; Kuo et al., 2000).

*I. pacificus* is established in at least 55 of California’s 58 counties and is commonly found in dense woodlands with a moist microclimate (Eisen et al., 2006, 2016; MacDonald, 2018). Moreover, multiple biotic and abiotic factors have determined contemporary and likely future suitable habitats for this tick (Hahn et al., 2016, 2021; Eisen et al., 2018). Seasonal variability in temperature, relative humidity, and precipitation may lead to increases in suitability over time (Hahn et al., 2016, 2021; Eisen et al., 2018). Temperature and relative humidity are important factors influencing tick density and may also influence pathogen infection in *I. pacificus* (Eisen et al., 2018, 2006, 2016).

Globally, 20 species of *Rickettsia* are known human pathogens, 16 of which are transmitted by ticks (Centers for Disease Control and Prevention [CDC], 2019). There are more tick-associated rickettsiae with undetermined pathogenicity being discovered episodically (Parola et al., 2013). Although the identities of other bacterial pathogens *I. pacificus* harbors have been well researched, studies on *Rickettsia* sp. carried by this tick are limited (Lane et al., 1981a; Lane et al., 1981b; Philip et al., 1981; Parola et al., 2013; Xu et al., 2019; Salkeld et al., 2021). For example, *Rickettsia tillamookensis* was first described from *I. pacificus* in 1976 and although shown to be mildly virulent in guinea pigs and deadly to mice, it remained unstudied until recently (Hughes et al., 1976; Gauthier et al., 2021; Paddock et al., 2022). *R. tillamookensis* was originally a member of the spotted fever group, but current molecular and phylogenetic analyses placed this species in a transitional group related to *R. asembonensis* and *R. felis* strain Pedreira, two species carried by cat fleas (Hughes et al., 1976; Gauthier et al., 2021; Maina et al., 2016, 2019).

In a 2011 study, *Rickettsia* species phylotype G022 (hereinafter, G022) and *Rickettsia monacensis* str. Humboldt (also known as *Rickettsia* species phylotype G021, abbreviated as strain Humboldt to differentiate it from the IrR/Munich strain of *R. monacensis* found in Europe) were first identified from adult *I. pacificus* ticks collected in California (Phan et al., 2011; Simser et al., 2002). In a follow up study, 2 % of adult *I. pacificus* tested positive for G022 and 100 % were found infected with strain Humboldt (Cheng et al., 2013a). Further studies showed transovarial and transstadial transmission of strain Humboldt was 100 %, and it possessed the ability to synthesize folate de novo, supporting the hypothesis that strain Humboldt is an endosymbiont (Cheng et al., 2013a, 2013b; Hunter et al., 2015). Preliminary phylogenetics placed G022 as a novel spotted fever group *Rickettsia*, with its capability to impact human health currently unknown (Phan et al., 2011).

Here, we examined the prevalence of G022 in *I. pacificus* nymphs and adults from multiple locations in California. This study represents the first investigation of G022 in *I. pacificus* nymphs. Additionally, nymphs were tested for *R. tillamookensis*. The prevalence of infection between and among collection sites were compared to determine if there were significant differences based on habitat type and climatic factors.

## 2. Materials and methods

### 2.1. Tick collections

**Fig. 1.**
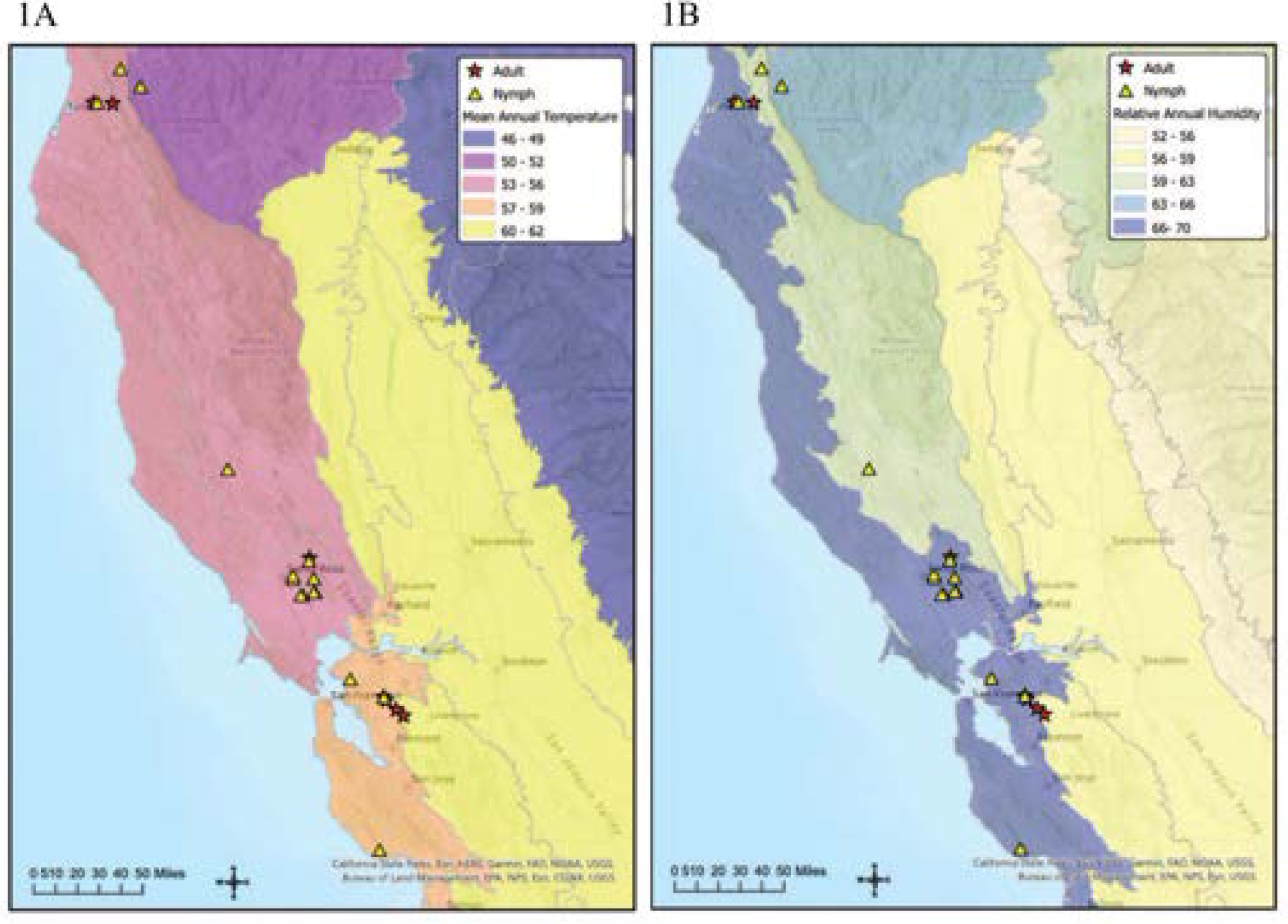
Collection-site map grouped by mean annual temperature in degrees Fahrenheit (1A) and collection map grouped by mean annual relative humidity, values expressed as percentages (1B). The samples spanned four different temperature zones: the 10–11.1 °C (50–52°F) zone, 11.7–13.3 °C (53–56°F) zone, 13.9–15 °C (57–59°F) zone, and 15.5–16.7 °C (60–62°F) zone. The samples spanned four different relative humidity zones: the 56–59 % range, 59–63 % range, 63–66 % range, and 66–70 % range. The most southern county sampled for ticks was Santa Cruz, with the most northern being Humboldt. Red stars designate the adult collection sites while yellow triangles indicate nymphal collection sites. The collection site in Napa County was the same for both adults and nymphs, so the icons overlap.

Location and vegetational data were recorded from each collection site and used to categorize the habitat type as montane hardwood conifer, coastal oak woodland, redwood, or annual grassland (Mayer and Laudenslayer, 1988). Location data from previously published research were used to determine where to collect questing ticks (Phan et al., 2011; Cheng et al., 2013a). *Ixodes pacificus* were collected in Alameda, Contra Costa, Humboldt, Mendocino, Napa, Santa Cruz, and Sonoma counties (Fig. 1 and Table 1) by dragging a 1-m^2^ white cotton flag over vegetation alongside wooded trails, in leaf litter, on tree trunks, logs, and rocks. Ticks were identified using key diagnostic characters presented in Furman and Loomis (1984).

Climatic data provided by the U.S. Forest Service’s ECOMAP 2007 and PRISM climate mapping system collaboration was used to assess any associations between prevalence and two climatic variables, mean annual temperature and mean annual relative humidity (USDA- Forest Service, 2017).

### 2.2. DNA extraction

Adult *I. pacificus* were kept alive and nymphs were stored in 70 % ethanol until processed. Ticks were cleaned in a series of bleach, ethanol, and water rinses and bisected using a sterile razor blade (Binetruy et al., 2019). Qiagen DNeasy Blood and Tissue kits (Germantown, MD) were used to extract DNA, with a total elution volume of 100 μL. For adults, half of the bisect was stored for future research. A mock extraction was included for each set of samples to be used as a negative control in quantitative polymerase chain reaction (qPCR).

### 2.3. Bacterial detection by qPCR

Ticks were tested for G022 following the qPCR protocol from Cheng et al. (2013a). The protocol was designed to amplify a 98-bp G022 *ompA* gene fragment. The testing conditions included a Taqman assay to detect the *ompA* amplicon using QuantStudio 3 (ThermoFisher, Carlsbad, CA). The CDC’s Rickettsial Zoonoses branch developed a protocol to amplify a 157-bp segment of the aspartate tRNA ligase gene, *aspS*, to determine *R. tillamookensis* prevalence in nymphs, with the testing conducted by their department (Paddock et al., 2022). The sequences of primers and probes are listed in Table 2.

For the qPCR experiment, the mock sample obtained during DNA extraction functioned as a negative control, while a non-template control substituted the tick DNA with water. The positive control for G022 was created by ligating the *ompA* gene into the StrataClone^™^ PCR cloning vector pSC-A (Agilent Technologies, La Jolla, CA). The positive control for *R. tillamookensis* was created by cloning the *aspS* gene using an Invitrogen TOPO TA cloning kit (Thermo Fisher Scientific, Carlsbad, CA). Since a standard for *R. tillamookensis* quantification was not used during testing, the cycle threshold (Ct) values were recorded instead. For qPCR, negative controls were run in triplicate, while the samples and standards were run in duplicate. Due to the lack of DNA concentration measurements before testing, qPCR results are reported as presence/absence without quantitation. Any samples showing amplification underwent a second round of qPCR, set up in the same manner. For both adult and nymphal samples, positive results were defined as having a Ct value below 40 with a DNA band at the expected size on gel electrophoresis for the G022 *ompA* gene, while no gel electrophoresis confirmation was done for the *R. tillamookensis aspS* gene. For the gel electrophoresis, 2 % TAE agarose gels containing 0.52 ug/mL ethidium bromide were made and visualized using an AlphaImager HP Imaging system (Alpha Innotech, San Leandro, CA).

### 2.4. Statistical analysis

A two-tailed Fisher’s exact test with Bonferroni correction utilizing tidyverse and rstatix programs in R studio was used to evaluate the association among collection sites, counties, habitat types, mean annual temperature, mean annual relative humidity, and G022 or *R. tillamookensis* presence (R Core Team, 2021). Any p-values less than 0.05 from the Fisher’s exact test, adjusted with Bonferroni correction, were further analyzed using a pairwise comparison for post-hoc evaluation. Another Fisher’s exact test was used to determine whether there is a statistically significant difference in G022 presence in adult versus nymphal *I. pacificus*. The coefficient of determination (R^2^) was calculated to look at the trends between the various conditions and G022 and *R. tillamookensis* prevalence. Wilson’s score 95 % confidence intervals (CI) were used while analyzing in RStudio (R Core Team, 2021). A 5 % significance value was used for all analyses.

## 3. Results

### 3.1. Adult and nymphal I. pacificus collections

A total of 720 adult *I. pacificus* was collected from 4 counties, 7 locations, and 3 habitats, while 550 nymphs were collected from 6 counties, 14 locations, and 3 habitats (Fig. 1). Collection sites, with specific numbers of ticks collected, spanned four habitat types (Table 1), four climatic zones based on the mean annual temperature range, and another four climatic zones based on the mean annual relative humidity (Fig. 1).

### 3.2. Prevalence of Rickettsia species phylotype G022 in I. pacificus adults

G022 was detected in adult ticks from all four counties. Out of 720 *I. pacificus* adults tested, 65 were positive by qPCR and gel electrophoresis, for an overall prevalence of 9.0 % (95 % CI = 7.2 %–11.3 %). Prevalence ranged from a low of 6.5 % in Humboldt County (95 % CI = 5.3 %–9.9 %) to a high of 16.7 % in Alameda County (95 % CI = 4.7 %–44.8 %) (Table 1). There was no statistically significant association between G022 infection in adults and any of the variables tested, which included county (R^2^: 0.94), individual collection sites within the county (R^2^: 0.99), habitat (R^2^: 0.86), mean annual temperature (R^2^: 0.99), and mean annual relative humidity (R^2^: 0.99) (Fisher’s exact, *p* > 0.05) (Tables 3 and 4).

### 3.3. Prevalence of Rickettsia species phylotype G022 in I. pacificus nymphs

Out of 550 nymphs, 29 were positive by qPCR and gel electrophoresis, for an overall prevalence of 5.3 % (95 % CI = 3.7 %–7.5 %). The prevalence ranged from a high of 12.3 % (95 % CI = 7.6 %–19.3 %) in Napa County to a low of 0 % in Santa Cruz County (95 % CI = 0 %–35.4 %). G022 positive nymphs were more likely to be found in Napa County compared to Contra Costa County (Fisher’s exact, *p* < 0.01), but no statistical significance was found when conducting pairwise comparisons between the other counties (R^2^: 0.28). There were not any statistically significant associations found between individual collection sites within the county (Fisher’s exact, *p* > 0.05, R^2^: 0.01) (Table 1).

When grouping the data of nymphs by habitat type, prevalence was 5.6 % (95 % CI = 3.9 %–7.9 %) in coastal oak woodland and 0 % prevalence in both redwood (95 % CI = 0 %–79.4 %) and montane hardwood conifer (95 % CI = 0 %–10 %). While positive nymphs were only found in coastal oak woodland, no statistically significant association was found between habitat type and G022 infection (Fisher’s exact, *p* > 0.05, R^2^: 0.99).

There was a statistically significant correlation between G022 prevalence and mean annual temperature (Fisher’s exact, *p* < 0.05, R^2^: 0.94) (Table 3). A pairwise comparison with Bonferroni correction showed a statistically significant outcome between the 11.7–13.3 °C (53–56°F) zone and the 13.9–15 °C (57–59°F) zone, with a higher likelihood of G022 positive nymphs in the 11.7–13.3 °C (53–56°F) zone than the 13.9–15 °C (57–59°F) zone (*p* < 0.05). The remaining pairwise comparisons, including those examining the association between G022 prevalence in nymphs and mean annual temperature zones or mean annual relative humidity (R^2^: 0.84), did not produce statistically significant results (Fisher’s exact, *p* > 0.05) (Table 4).

Comparing the prevalence of G022 in nymphs versus adults also led to a statistically significant result (Fisher’s exact, *p* < 0.05). Overall, G022 positive nymphs were less likely to be found compared to G022 positive adults (OR, 0.60; 95 % CI = 36.5 %–95.1 %, R^2^: 0.99).

### 3.4. Prevalence of R. tillamookensis in I. pacificus nymphs

Due to low sample volume, DNA samples from Sonoma and Santa Cruz counties were not screened for *R. tillamookensis*. Out of 440 nymphs tested, seven were qPCR positive, for an overall prevalence of 1.6 % (95 % CI = 0.8 %–3.3 %). The prevalence ranged from a high of 4.9 % (95 % CI = 1.4 %–16.1 %) in Humboldt County to a low of 0.82 % (95 % CI = 0.1 %–4.5 %) in Napa County (Table 1). Analysis using Fisher’s exact test indicated a lack of statistically significant association between *R. tillamookensis* infection in nymphs and either the county (R2: 0.08) or individual collection sites within the county (R^2^: 0.02) (Fisher’s exact, *p* > 0.05).

When looking at the data by habitat type, *R. tillamookensis* had a prevalence of 6.9 % in montane hardwood conifer (95 % CI = 1.9 %–22.0 %), 1.2 % (95 % CI = 0.5 %–2.8 %) in coastal oak woodland, and 0 % (95 % CI = 0 %-79.4 %) in redwood. Statistical analysis determined that there was no statistically significant correlation between *R. tillamookensis* infection in nymphs and habitat type (R^2^: 0.90), mean annual temperature (R^2^: 0.97), or mean annual relative humidity (R^2^: 0.47) (Fisher’s exact, *p* > 0.05) (Tables 3 and 4). Finally, none of the nymphs tested by qPCR were found to be coinfected with both G022 and *R. tillamookensis*.

## 4. Discussion

*Rickettsia* species phylotype G022 and *R. tillamookensis*-infected *I. pacificus* nymphs were detected in multiple Northern California counties at an overall prevalence of 5.3 % and 1.6 %, respectively. G022-positive nymphs were detected in all but one county (Santa Cruz) while *R. tillamookensis* was detected in nymphs collected and tested from four counties. Comparison of G022 prevalence between adult and nymphal *I. pacificus* revealed that nymphs were less likely than adults of being infected with G022. The prevalence of G022 in adults vs nymphs varied significantly between Humboldt County (6.5 % in adults, 2.4 % in nymphs) and Contra Costa County (11.7 % in adults, 0.9 % in nymphs), compared to Napa County, where the prevalence was similar for both adults (12.9 %) and nymphs (12.3 %). This highlights distinct prevalence patterns between adult and nymphal stages in different collection areas.

G022 was originally identified in 2011 from *I. pacificus* adult ticks collected from the Napa Valley (Phan et al., 2011). Adult ticks in the current study were collected from counties that had G022 positive ticks in previous studies to increase the likelihood of finding additional positive ticks. Due to travel restrictions during the Covid-19 pandemic, most *I. pacificus* adults were collected from redwood habitat at the Green Diamond Resource Co. in Humboldt County. The calculated prevalence of G022 in adults in this study was 9.0 % (95 % CI = 7.2 %–11.3 %) overall, higher than the previous study’s 2 % (Cheng et al., 2013a) and similar to the prevalence of other well-known TBPs (Salkeld et al., 2021; Eshoo et al., 2015; MacDonald et al., 2017; Saunders and Kjemtrup, 2022; Holden et al., 2003; Lane et al., 2007, 2010; Sambado et al., 2020). The variation in G022 prevalence among adult ticks observed between this study and previous studies may be attributed to differences in collection locations and the larger number of ticks sampled from a single county. Humboldt County, with a prevalence of 3.2 % in adult *I. pacificus*, recorded the second-highest G022 prevalence among the seven counties surveyed, compared to the average prevalence of 2.0 % reported in 2013 (Cheng et al., 2013a).

Analyzing adult prevalence data by county, habitat, mean annual temperature, and mean annual relative humidity yielded no statistically significant correlations. Most specimens were collected either on walking trails or adjacent to them, so the chance of human encounters is high. Dense oak woodlands pose a higher risk for *I. pacificus* encounters in Southern and Northern California (MacDonald et al., 2017; Lane et al., 2007; Eisen et al., 2003, 2009, 2010). Apart from being present in oak woodland habitat, coastal chaparral was found to be another habitat for potentially acquiring tick-borne disease agents (Salkeld et al., 2021).

The prevalence of *R. tillamookensis* was 1.6 % (95 % CI = 0.8 %–3.3 %) in nymphal *I. pacificus*, which is similar to results of a recent study (Paddock et al., 2022). It is unsurprising that we detected G022 in *I. pacificus* nymphs since many rickettsiae are passed transstadially from one parasitic life stage to the next (Azad et al., 1998; Macaluso et al., 2002; Bonnet et al., 2017). Detecting G022 in questing nymphs indicates transstadial transmission from the larval stage. The overall prevalence was a modest 5.3 %, and all infected nymphs were derived from coastal oak woodlands. On the other hand, some significant differences were detected when the prevalence of G022 was compared among counties and mean annual temperatures. These findings could have future ramifications as climatic changes may reduce infection rates in certain areas while increasing them in others as habitat suitability shifts (Eisen et al., 2018, 2016; Padgett and Lane, 2001).

Analyses of mean annual temperature and mean annual relative humidity did not reveal significant associations between G022 prevalence and adult *I. pacificus*. There was a statistically significant association between the prevalence of G022 in *I. pacificus* nymphs and mean annual temperature, but *R. tillamookensis* infection was not correlated with either variable. The 11.7–13.3 °C (53–56°F) zone had an increased likelihood of having G022 positive nymphs than the 13.9–15 °C (57–59°F) zone. These ranges coincide with some inland areas north of San Francisco and the San Francisco Bay Area, respectively.

Since ticks spend more than 90 % of their life cycles off-host, environmental conditions impact their development and survival. In the San Francisco Bay Area, for example, *I. pacificus* females oviposited at temperatures ranging between 6.5 °C and 29 °C, whereas egg development proceeded within a narrower range spanning 9 to 25 °C and hatching success was lower in exposed sites versus sheltered plots (Peavey and Lane, 1996).

One reason why there was a significant association between G022 in *I. pacificus* nymphs and temperature that was not seen in adults or with *R. tillamookensis* could be due to the climate sections map overlay that was used instead of recording exact microclimatic temperature measurements during each collection trip. While the initial temperature was taken on all collection trips, it was not as precise a method as previous studies that measured temperature at the soil surface (Lane et al., 2007; Padgett and Lane, 2001; Peavey and Lane, 1996). Moreover, relative humidity was not logged on field trips. Adopting the use of temperature and relative humidity data loggers (Lane et al., 2007) to track diurnal changes during collections would increase accuracy, thereby elucidating how microclimatic conditions may influence pathogen prevalence in *I. pacificus*.

## 5. Conclusions

Our study found that nymphal *I. pacificus* can be infected with G022, albeit at a lower prevalence than adults (nymphs: 5.3 %). Moreover, the detection of G022 in questing nymphs and adult ticks suggests that it can be passed transstadially by *I. pacificus* larvae and nymphs. The prevalence of *R. tillamookensis* in nymphal *I. pacificus* is consistent with previous findings (1.6 %). The low prevalence of G022 and *R. tillamookensis* among adults and nymphs suggests horizontal transmission occurs between ticks and their vertebrate hosts for these *Rickettsia* species. Despite the high sensitivity of qPCR, false negatives were possible if the DNA concentration in a given tick extract was too low. Further studies are needed to determine the vertebrate host and their pathogenicity to humans. This study also preliminarily investigated whether two climatic variables, mean annual temperature and mean annual relative humidity, may influence the prevalence of the *Rickettsia* species in *I. pacificus*. The only significant outcome was G022 infected nymphs had a higher likelihood of being in the 11.7–13.3 °C zone than 13.9–15 °C, which merits further study. Of six counties surveilled, Napa County had the highest prevalence of G022 positive nymphs, whereas Humboldt County had the highest prevalence of *R. tillamookensis*-positive nymphs. A potential limitation of this study was that it may have been underpowered due to the relatively low numbers of ticks collected from the various locations.

## Figures and Tables

**Table 1:** Prevalence data for *Rickettsia* species phylotype G022 and *R. tillamookensis* in *Ixodes pacificu*s adults and nymphs by location. All adults were collected in 2020 and nymphs are from multiple years.

County	Location	Coordinates	Date Collected	Habitat	No. of Adults (%, 95 % CI) with G022/n	No. of Nymphs (%, 95 % CI) with G022/n	No. of Nymphs (%, 95 % CI) *with R. tillamookensis/ n*

Alameda	Dublin hills	37.718055, - 121.914722	3/19/20, 3/31/20	Annual Grassland	2(16.7 %,4.7 %- 44.8 %)/12	N/A	N/A
Contra Costa	Las Trampas Regional Wilderness	37.815511, - 122.047095	1/3/20, 4/27/21, 5/ 16/21	Oak Woodland	5(12.8 %,5.6 %- 26.7 %)/39	1(1.5 %, 0.3 %- 8.1 %)/66	0(0 %, 0 %- 6.0 %)/66
	Bishop Ranch Regional Preserve	37.75, - 121.964166	4/13/20	Oak Woodland	0(0 %, 0 %- 40.0 %)/5	N/A	N/A
	Bollinger Canyon Rd.	37.798349, - 122.029860	2/21/20, 3/10/20	Oak Woodland	4(12.1 %, 4.8 %- 27.3 %)/33	N/A	N/A
	Tilden Regional Park	37.910555, - 122.267222	5/10/21	Oak Woodland	N/A	0(0 %, 0 %- 8.0 %)/46	1(2.2 %, 0.4 %- 11.3 %)/46
	County Prevalence	N/A	N/A	N/A	9(11.7 %,6.3 %- 20.8 %)/77	1(0.9 %, 0.2 %- 4.9 %)/112	1(0.9 %, 0.2 %- 4.9 %)/112
Humboldt	Green Diamond Resource Co. - MR4100	40.863611, - 123.970277	5/4/20, 5/15/20, 5/ 21/20, 6/3/20, 6/8/ 21	Redwood	31(7.4 %,5.3 %- 10.4 %)/417	0(0 %, 0 %- 80.0 %)/1	0(0 %, 0 %- 80.0 %)/1
	Green Diamond Resource Co. - Fulton Ranch	40.859166, - 123.843888	5/23/20	Redwood	4(6.0 %, 2.4 %- 14.4 %)/67	N/A	N/A
	Lacks Creek Management Area	41.033888, - 123.791388	6/23/21, 6/27/21, 7/ 3/21	Montane Hardwood Conifer	N/A	0(0 %, 0 %- 10.0 %)/29	2(6.9 %, 1.9 %-22.0 %)/29
	Six Rivers National Forest - Boise Creek Campground	40.944999, - 123.657777	7/21/21, 7/29/21	Oak Woodland	N/A	1(9.0 %, 1.6 %- 37.7 %)/11	0(0%, 0%-29.5%)/11
	County Prevalence	N/A	N/A	N/A	35(6.5 %,5.3 %- 9.9 %)/538	1(2.4 %, 0.43 %- 12.6 %)/41	2(4.9%,1.4%- 16.1%)/41
Mendocino	Hopland Research and Extension Center (HREC)	39.000556, - 123.079721	5/24/09	Oak Woodland	N/A	5(3.0 %, 1.3 %- 6.9 %)/165	3(1.8 %, 0.6 %- 5.2 %)/165
Napa	Bothe - Napa Valley State Park	38.540939, - 122.537876	1/10/20, 4/28/21	Oak Woodland	19(12.9 %,7.6 %- 19.3 %)/147	15(12.3 %,7.6 %- 19.3 %)/122	1(0.8 %,0.1 %- 4.5 %)/122
Santa Cruz	Forest Ecology Research Plot (FERP)	37.012400, - 122.075000	10/21/16, 10/24/16	Oak Woodland	N/A	0(0 %, 0 %- 40.0 %)/7	N/A
Sonoma	Sugarloaf State Park	38.436885, - 122.510293	5/31/07	Oak Woodland	N/A	0(0 %, 0 %- 50.0 %)/4	N/A
	Bouverie State Park	38.367777, - 122.513055	4/20/06	Oak Woodland	N/A	3(33.3 %, 10 %- 60.0 %)/9	N/A
	Annadel State Park	38.434444, - 122.632538	5/15/07, 5/17/07, 5/ 21/07	Oak Woodland	N/A	0(0 %, 0 %- 10.0 %)/31	N/A
	Fairfield Osborne Preserve	38.343333, - 122.594722	4/27/06, 5/23/07, 5/ 24/07, 5/28/19	Oak Woodland	N/A	4(12.5 %,5.0 %- 28.1 %)/32	N/A
	Spring Lake Regional Park	38.451111, - 122.650277	5/29/19, 5/22/20	Oak Woodland	N/A	0(0 %, 0 %- 20.0 %)/16	N/A
	Audobon Bouverie Preserve	38.364359, - 122.509971	5/22/07 to 6/1/07	Oak Woodland	N/A	0(0 %, 0 %- 29.5 %)/11	N/A
	County Prevalence	N/A	N/A	N/A	N/A	7(6.7 %, 3.3 %- 13.4 %)/103	N/A
Total					65(9.0 %,7.2 %- 11.3 %)/720	29(5.3 %,3.7 %- 7.5 %)/550	7(1.6 %, 0.8 %- 3.3 %)/440

N/*A*= no samples from location tested, *n*= the number of ticks collected.

1 N/A under Coordinates, Date collected, and Habitat is a placeholder for empty space.

**Table 2:** Primers and probes used in the real-time PCR assay.

Gene name	Primer/Probe	Sequence (5′ to 3′)	PCR Product Size	Reference

*ompA* (phylotype G022)	22-F	CTGCAGATATAGCCGGTCGTATT	98-bp	Cheng et al. (2013a)
*ompA* (phylotype G022)	22-R	TAATCGAACCAACGACGGTATTT		Cheng et al. (2013a)
*ompA* (phylotype G022)	22-probe	VIC—CTCCCGTAGGTCTAAA-MGB		Cheng et al. (2013a)
*ompA* (*R. monacensis* str. Humboldt)	21-F	ACGGCTGGAGGAGTAGCTAATG	76-bp	Cheng et al. (2013a)
*ompA* (*R. monacensis* str. Humboldt)	21-R	GATTACCACCGTAAGTAAATGCCTTAT		Cheng et al. (2013a)
*ompA* (*R. monacensis* str. Humboldt)	21-probe	FAM-TCCTGTTGACGGTCCT-MGB		Cheng et al. (2013a)
*aspS* (*R. tillamookensis*)	TillF4	GCTTGCTGATTTAAAGGAAATGCA	157-bp	Paddock et al. (2022)
*aspS* (*R. tillamookensis*)	TillR4	AGGAAGTTGATAAGAGATTTGGGG		Paddock et al. (2022)
*aspS* (*R. tillamookensis*)	Till_P	FAM-CCATGCGGCGGTGCTCCAAACT-BHQ1		Paddock et al. (2022)

**Table 3:** Prevalence of *Rickettsia* species phylotype G022 and *R. tillamookensis* based on mean annual temperature range.

Mean Counties’ Annual Temperature Range ( °C)	County	Location	G022 Prevalence in Adults (95 % CI)/n	G022 Prevalence in Nymphs (95 % CI)/n	*R. tillamookensis* Prevalence in Nymphs (95 % CI)/n

10–11.1	Humboldt	Boise Creek	N/A	9.0 % (1.6 %– 37.7 %)/11	0 % (0 %– 25.9 %)/11
11.7–13.3	Humboldt	Green Diamond	8.6 % (6.6 %– 11.0 %)/631	*6.4 % (4.5 %– 9.2 %)/420	1.9 % (0.9 %– 4.1 %)/317
		Resource Co.			
		Lacks Creek			
		Recreation Area			
	Napa	Bothe			
	Mendocino	HREC			
	Sonoma	See Table 1			
13.9–15	Contra	Las Trampas	12.5 % (6.3 %– 20.8 %)/72	*0.8 % (0.2 %– 4.6 %)/119	0.9 % (0.2 %– 4.9 %)/112
	Costa	Bollinger Canyon^1^			
		Tilden Regional Park			
	Santa Cruz	FERP			
15.5–16.7	Alameda	Dublin Hills	11.8 % (4.7 %– 44.8 %)/17	N/A	N/A
	Contra	Bishop Ranch			
	Costa				

N/*A*= no sample in that range.

* statistically significant difference between two ranges.

1 location specific to adult results.

2 Prevalence data is given per grouping of mean temperature range.

**Table 4:** Prevalence of *Rickettsia* species phylotype G022 and *R. tillamookensis* based on mean annual relative humidity range.

Mean Annual Relative Humidity (%)	County	Location	G022 Prevalence in Adults (95 % CI)/n	G022 Prevalence in Nymphs (95 % CI)/n	*R. tillamookensis* Prevalence in Nymphs (95 % CI)/n

56–59	Alameda	Dublin Hills	11.7 % (3.3 %– 34.3 %)/17	N/A	N/A
	Contra	Bishop Ranch			
	Costa				
59–63	Humboldt	Green Diamond- Fulton	6.0 % (2.4 %– 14.4 %)/67	2.6 % (1.1 %– 5.9 %)/194	2.6 % (1.1 %– 5.9 %)/194
		Ranch^1^			
		Lacks Creek			
	Mendocino	HREC			
63–66	Humboldt	Boise Creek	N/A	9.1 % (1.6 %– 37.7 %)/11	0 % (0 %– 25.9 %)/11
66–70	Napa	Bothe	9.3 % (7.3 %– 11.8 %)/636	6.7 % (4.5 %– 9.8 %)/345	0.9 % (0.2 %– 3.1 %)/235
	Humboldt	Green Diamond-MR4100			
	Contra	Las Trampas			
	Costa	Tilden Regional Park			
		Bollinger Canyon^1^			
	Sonoma	See Table 1			
	Santa Cruz	FERP			

N/*A*= no sample in that range,

1 location specific to adult results.

3 Prevalence data is given per grouping of mean relative humidity range.

## Data Availability

Data will be made available on request.
